# The Impact of Obesity, Adipose Tissue, and Tumor Microenvironment on Macrophage Polarization and Metastasis

**DOI:** 10.3390/biology11020339

**Published:** 2022-02-21

**Authors:** Ola Habanjar, Mona Diab-Assaf, Florence Caldefie-Chezet, Laetitia Delort

**Affiliations:** 1 Université Clermont-Auvergne, INRAE, UNH, ECREIN, f-63000 Clermont-Ferrand, France; ola.habanjar@etu.uca.fr (O.H.); florence.caldefie-chezet@uca.fr (F.C.-C.); 2 Equipe Tumorigénèse Pharmacologie moléculaire et anticancéreuse, Faculté des Sciences II, Université libanaise Fanar, Beyrouth 1500, Liban; mdiabassaf@ul.edu.lb

**Keywords:** extracellular matrix, tumor microenvironment, cancer-associated fibroblasts, metastasis, immune-inflammatory cells, cancer-associated adipocytes, tumor-associated macrophages, macrophage polarization, angiogenesis, hypoxia

## Abstract

**Simple Summary:**

The inflammatory adipose microenvironment in obesity plays a crucial role in cancer development and metastases. By focusing on adipocytes and macrophages, as well as the extracellular matrix, the cellular and molecular mechanisms that link inflammation, obesity, and cancer will be addressed by this review. After describing the tumor microenvironment and extracellular matrix, the influence of M1, M2, and tumor-associated macrophages will be explored through their origin, classification, polarization, and regulatory networks, including their potential role in angiogenesis, invasion, metastasis, and immunosuppression, with a specific focus on the roles of adipocytes in this process.

**Abstract:**

Tumor metastasis is a major cause of death in cancer patients. It involves not only the intrinsic alterations within tumor cells, but also crosstalk between these cells and components of the tumor microenvironment (TME). Tumorigenesis is a complex and dynamic process, involving the following three main stages: initiation, progression, and metastasis. The transition between these stages depends on the changes within the extracellular matrix (ECM), in which tumor and stromal cells reside. This matrix, under the effect of growth factors, cytokines, and adipokines, can be morphologically altered, degraded, or reorganized. Many cancers evolve to form an immunosuppressive TME locally and create a pre-metastatic niche in other tissue sites. TME and pre-metastatic niches include myofibroblasts, immuno-inflammatory cells (macrophages), adipocytes, blood, and lymphatic vascular networks. Several studies have highlighted the adipocyte-macrophage interaction as a key driver of cancer progression and dissemination. The following two main classes of macrophages are distinguished: M1 (pro-inflammatory/anti-tumor) and M2 (anti-inflammatory/pro-tumor). These cells exhibit distinct microenvironment-dependent phenotypes that can promote or inhibit metastasis. On the other hand, obesity in cancer patients has been linked to a poor prognosis. In this regard, tumor-associated adipocytes modulate TME through the secretion of inflammatory mediators, which modulate and recruit tumor-associated macrophages (TAM). Hereby, this review describes the cellular and molecular mechanisms that link inflammation, obesity, and cancer. It provides a comprehensive overview of adipocytes and macrophages in the ECM as they control cancer initiation, progression, and invasion. In addition, it addresses the mechanisms of tumor anchoring and recruitment for M1, M2, and TAM macrophages, specifically highlighting their origin, classification, polarization, and regulatory networks, as well as their roles in the regulation of angiogenesis, invasion, metastasis, and immunosuppression, specifically highlighting the role of adipocytes in this process.

## 1. Introduction

Cancer is typically associated with neo-angiogenesis, tumor-enhanced inflammation, and the uncontrolled growth of abnormal cells. Ultimately, malignant tumors invade normal tissue and spread throughout the body [1,2,3]. The extent of a tumor invasion is a key index of cancer severity. Highly invasive tumor cells gain access to blood vessels by leaving the primary tumor site and invading the surrounding tissues, reaching the blood and lymphatic vessels where they disseminate to remote organs [4,5,6,7]. The metastasis of these cancers accounts for approximately 90% of cancer-related deaths [8,9]. Historically, the search for metastasis began in 1889 by surgeon Stephen Paget, who hypothesized the concept of “seed and soil.” This concept describes the dependence of cancer metastasis on tumor cells (seeds) and its complex interactions with the tumor microenvironment (TME) (soil) [10,11,12]. In fact, a non-neoplastic microenvironment can prevent tumor invasion and metastasis. The TME guides tumor scaffolds with new cell interactions to form a pre-metastatic niche (PMN). Generally, the PMN consists of (i) cellular components such as myofibroblasts, fibroblasts, adipocytes, and immune-inflammatory cells, and (ii) non-cellular components of tumor niches such as extracellular matrix (ECM) including the blood and lymphatic networks [13].

The stroma is generally rich in active biomolecules such as growth factors (GFs) and cytokines. However, TME cells can regulate the behavior of tumor cells to strengthen or suppress the tumor [14,15,16]. Among these cells are macrophages, which make up to 50% of the mass of most tumors and produce an inflammatory microenvironment [17,18,19]. Inflammatory interventions in tumor metastases were first described by Virchow in 1867 [20]. Since then, many studies have identified the pathways and types of cell-cell and cell-matrix interactions. Macrophages are the most common inflammatory cells recruited towards the tumor site. They are present at all stages of tumor progression [21]. Leek and Harris described macrophages as the “Swiss Army knife” of the immune system [22], as they are involved in the regulation of tissue homeostasis, the inflammatory response to pathogens, and wound healing [23,24]. These cells show high phenotypic heterogeneity (different subpopulations) depending on the microenvironment [25,26]. It is possible to distinguish phenotypic M1 (inflammatory), M2 (neoplastic), and tumor-associated macrophages (TAMs). In primary tumors, macrophages can stimulate angiogenesis and improve tumor cell motility and infiltration. Then, during metastasis, they initiate the pre-metastatic site, promoting extravasation, survival, and the sustained growth of tumor cells. Macrophage polarization towards a tumor-promoting phenotype is due not only to the disruption of tissue homeostasis, but also to the interaction between malignant and stromal cells in TME [27]. Tumor initiation, progression, and metastasis are affected by dynamic changes in macrophage phenotypes. There is increasing evidence that defined subpopulations of macrophages are involved in these tumor-promoting activities. Metastases include hypoxia, angiogenesis (formation of a complete vasculature), and even overweight and obesity, as studies have shown a positive link between BMI and postmenopausal breast cancer risk [28]. Obesity increases the risk of death in both premenopausal and postmenopausal patients with breast cancer, but the effects of obesity on the risk of breast cancer in premenopausal (lower risk of ER-positive breast cancer) and postmenopausal (markedly higher risk of ER-positive breast cancer) women differ based on estrogen receptor (ER) status [29,30].

Possible interactions between the metabolism and the immune system in obese patients are currently a new area of research. Chronic low-grade inflammation is closely associated with obesity, which is closely associated with metastasis [31]. More specifically, obesity-related fatty inflammation results from a pro-inflammatory dialogue between adipocytes and immune cells. Adipocytes in obese people are thought to be immunocompetent cells that play a direct immunological role in antigen presentation, T cell polarization (activated Th1), adipose tissue macrophage (ATM) recruitment, and M1 polarization [32,33,34]. Therefore, obesity/overweight (adipocytes), inflammation (macrophages), and tumors (transformed cells) form an important triangle that is proven to regulate TME [35,36,37].

## 2. Tumor Microenvironment

An ECM is a microenvironment with complex biomechanical and physico-chemical properties. This structure provides a platform for cell growth and tissue homeostasis in all organs [38,39,40,41]. Histologically, it is composed of the following two major parts: (i) the structural interstitial matrix surrounding the cells (collagen I and fibronectin FN) and (ii) the basement membrane (collagen IV, VIII, X, and laminins) [42,43,44,45,46]. Thanks to the masking of receptors by collagen, there are various proteins (collagen, FN), polysaccharides, GF-rich local deposits, and inactive bioactive molecules (metabolic precursors) [47,48,49,50,51,52,53]. Collagen (fibrotic and non-fibrotic) is the most abundant structural protein in human tissue, accounting for about 30% of the total protein content of the body [43,46,54,55,56]. It regulates adhesion, cell migration [56], and tensile strength to maintain homeostasis [57]. Basically, fibroblasts secrete interstitial collagen, FN, and metalloproteinases (MMPs) that degrade the ECM and most of the GFs that promote differentiation, growth, and cell migration [58,59,60]. FN binds these cells to the ECM [46], and thus forms a fibrous network [61]. It is involved in the organization of interstitial ECM through interaction with type I collagen [61,62,63,64,65] and in developing cell migration and tumor metastasis [66]. When cancer develops, various disorders appear in this matrix, exacerbating tumor progression. In general, this small disruption of orientation affects homeostasis and promotes malignant growth [67,68], but the proper composition is sufficient to contain it [69,70]. Tumor cells cooperate with inflammatory cells to form PMNs that are beneficial for cancer progression [71]. The field of study has evolved over the years, explaining the role of primary tumors in altering the microenvironment to produce growth-favorable PMNs [12,72,73,74]. This PMN, in turn, promotes tumor growth under the effects of increased inflammation, vascular permeability, immunosuppression, and tissue rigidity [75,76,77]. As cancer grows rapidly, various dynamic interactions occur between these microenvironments, cancer cells, and resident cells [78,79,80]. It is important to note that the cooperation of transformed tumor cells and macrophages results in delayed cell-microenvironment interaction, cell proliferation, differentiation, and apoptosis [50,81,82]. Macrophages and tumor cells secrete various cytokines, chemokines, hormones, MMPs, Lysyl Oxidases (LOX: enzymes secreted by primary tumor cells for linear cross-linking of collagen) and GFs, which are initially interstitial. Such interaction activates fibroblasts and induces cancer-associated fibroblasts (CAFs) [40,83]. After activation, CAFs increase the regulation of FN, collagens I, III and IV, elastin, GFs (such as VEGF, which promotes vascular permeability, angiogenesis, and metastasis), and immunosuppressive cytokines (such as interleukin (IL)-22) [14,15,16,79,84,85,86,87,88,89,90]. This neosynthesized collagen deposition will be reoriented and cross-correlated primarily by LOX to form more rigid and large-aligned fibrils [70,91,92,93]. As a result, the linearization of collagen fibers, located along the edge, facilitates the invasion and tumor metastasis of neoplastic cells [94,95]. The alteration of the homeostatic balance is likely to create a “reactive” stroma that promotes a successive transition of transformed cells to invasive cancer, either by excessive production of GFs or through reciprocal interactions between stromal and tumor cells [96].

On the other hand, the adipose matrix influences TME. Generally, adipose tissue is complex and dynamic. It is composed mostly of white adipose tissue (WAT) containing adipose stem cells (ASC), immune cells (macrophages, lymphocytes), and endothelial cells [97]. It can create an adipose microenvironment rich in adipokines, estrogens, GFs, and proliferating hormones that surround the tumor [98,99]. Adipocytes, which can differentiate into numerous cell types, may function as endocrine and inflammatory cells. They contain a powerful energy depot (fatty acid) and, conversely, can secrete metabolic substrates, lipid agonists (loose fatty acid), pro-inflammatory molecules, numerous hormones, adipokines, GFs, and cytokines. Such activities set off both the persistent low-grade inflammation in adipose tissue related to weight problems and most cancer proliferation, migration, and remedy resistance [32,33,34,98,100,101,102,103]. Actually, the above-average proportion of adipose tissue in body weight (overweight or obesity) may be associated with an imbalance of many cells and hormonal metabolic pathways that increase the risk of most cancers [104] through reciprocal feedback between adipocytes and tumor cells [35,98,105,106,107]. Adipose cells secrete approximately 50 exclusive cytokines, chemokines, and hormonal elements including IL-6, IL-8, IL-1β, tumor necrosis factor (TNF)-α, VEGF, CCL2, CCL5, and MMP9, which can also make a contribution to obesity and TME. Reciprocally, most cancer cells in this adipose matrix secrete diverse cytokines, which expand the formation of its PMN [37,108,109,110]. Elucidating the interaction between cancer-associated adipocytes (CAAs) in the TME and that between the TME and the adipose microenvironment (in obese or overweight individuals) during tumor invasion and progression is the focus of much investigation [111].

## 3. Degradation of ECM and Metastases

Biologically, tumor cells, CAFs, macrophages (TAM and M2), and CAAs are involved in the degradation of the ECM by MMP secretion [37,108,109,110,112,113,114,115]. MMPs are proteolytic enzymes that destroy the ECM surrounding the tumor, allowing tumor cells to escape the primary tumor and invade the surrounding tissue, thus enhancing metastases [84,113,116,117]. MMPs are normally secreted as zymogens (inactive) and activated in the extracellular space [118]. MMP’s involvement in the ECM collagen degradation releases adhesion sites and targets receptors for activation of GF [59,119,120,121,122]. It also provides exposure of matrix signaling components [81,123]. Changes in biochemical composition and degradation of the basement membrane lead to changes in intracellular signaling and tumor spread [124,125]. These interactions generate positive counter-regulations that re-stimulate tumor growth and survival [14,15,89]. It can, therefore, be hypothesized that CAF is a major effector that contributes to the metastatic pro-inflammatory state [84,126], ECM remodeling, angiogenesis [127,128,129,130,131], perturbation of tissue homeostasis, ECM stiffness, and the stimulation of malignant progression by enhancing GF signaling [15,70,132,133]. Because the inhibition of MMPs may limit metastases, the role of MMPs in tumor invasion has attracted the attention of researchers in cancer treatment [134,135,136,137]. Metastasis is one of the main hallmarks of most malignant solid tumors, and research has already revealed several hypotheses that may explain the stimuli of metastasis phenomena. There are physical and natural barriers at the boundary between the epithelial cells and the matrix. In the case of cancer, these barriers interfere with the invasion of cancer cells and their attachment to the ECM. Such barriers may be related to the myoepithelial cell layer [138,139]. Tumor cell progression usually begins with a transformation of these barriers that can be disrupted (by MMPs) and/or altered (by invadopods, which are actin-rich, convex subcellular structures) for tumor cell migration. Metastases are further induced by ASCs, which can differentiate into other types of cancer-associated cells. Cancer-associated adipocytes (CAA) also secrete various cytokines, chemokines, hormonal factors, and MMPs (MMP-9) that enhance TME interactions and promote matrix degradation and metastasis [37,108,109,110,140,141,142,143,144].

The disappearance of these barriers is then dependent on several stimuli [138,139,145,146,147,148,149,150,151]. For example, mechanical stress induces a mechanical force that is managed by the rapid accumulation of cancer cells on the barrier layer. Cracks in the basement membrane are managed by cooperating with organisms that invade cancer cells. Invadopods, which initially initiate a convex branch, then expand to extend the cellular membrane and allow cancer cells to pass through the primary site to the stroma, thus promoting invasive anchoring. These invaders disrupt nearby healthy tissues because laminin and collagen IV are removed rather than completely broken down.

**Figure 1:**
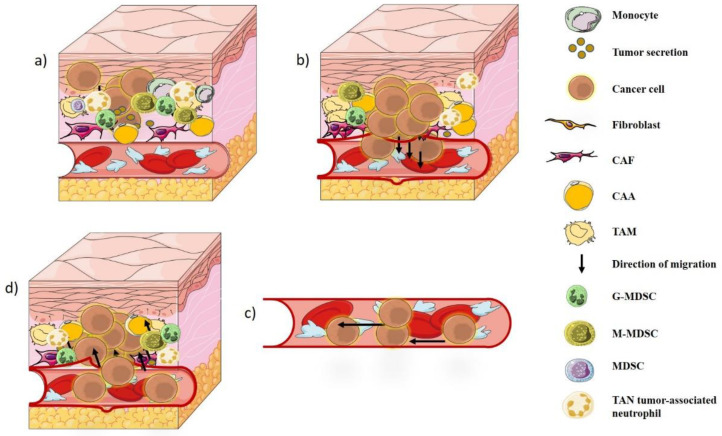
Different stages of cancer metastasis: (**a**) Invasion of primary sites, (**b**) Invasion into the vascular system, (**c**) Survival within the circulation, (**d**) Extravascular extravasation, adaptation, and growth in the metastatic niches.

Once this barrier is impaired, tumor migration begins. The cancerous cells have the ability to cross the basement membrane from the primary sites, where they originate, to distant target sites via lymphatic vessels and/or blood circulation [4,5,6,7]. The epithelial–mesenchymal transition (EMT) of differentiated epithelial cells is then characterized by the loss of epithelial cell properties and the acquisition of a new migratory mesenchymal phenotype [152]. In fact, cancer cells subjected to EMT (elongated form) can then migrate [153,154]. Since TME is an obligatory inducer of cancer growth [76,155], the obligatory stages of tumor metastasis, known as the metastatic flow, are generally as follows: (a) Invasion of primary sites, (b) Invasion into the vascular system, (c) Survival within the circulation, (d) Extravascular extravasation and adaptation and growth in the metastatic niche [156,157,158]. Adipocytes, immune cells, and mediators of the inflammatory response may directly contribute to tumor growth and metastasis [159,160,161,162,163] (Figure 1). CAAs and several M2 and TAM macrophages secrete different soluble factors, such as IL-6, IL-8, IL-1β, TNF-α, and TGF-β that participate in EMT to promote invasion [144,164,165,166,167]. In brief, the adipose matrix promotes interactions between adipocytes, vascular stromal cells, cancer cells, and macrophages during metastasis [168].

## 4. Tumor–Immune Interaction

Phagocyte stimulation, a key step in immunity, was predicted by the Nobel Prize-winning Elie Metchnikoff in 1908 [169]. Macrophages are the first line of defense against infection [170,171], as they resolve acute inflammation [172], remove cell and apoptosis debris [173], regulate tissue stress and metabolic responses to apoptosis, and they intervene with the TME [174,175,176]. The first discovery of the presence of immune cells in human tumors was made by Virchow in 1863 [20]. This has led immunologists to investigate macrophages as immunophagocytic cells and effectors in most malignancies [177,178]. The density of macrophages in tumors increases with tumor progression [179,180,181]. Macrophages are not usually present in non-tumor tissues [182,183]. These cells are derived from the differentiation of the mononuclear phagocyte lineage. Peripheral blood monocytes eventually leave the blood circulation and are mobilized from the local circulation to the tumor where they differentiate into TAM [22,159,184,185]. TAMs and CAAs are important orchestrators for associating inflammation with cancer progression. Their differentiation is stimulated by chemotactic factors secreted by cancer cells [186,187,188] to promote tumor progression such as proliferation induction, ECM remodeling, angiogenesis, and adaptive immune evasion [189,190,191], either by the release of EGF [192], or by the degradation of ECM proteins (MMP2 and MMP9) [37,108,109,110,140,141,142,143,144,193,194,195]. Next, the high cytokine and chemokine levels released by monocytes, macrophages, tumor cells, fibroblasts, endothelial cells, and TAMs are key mediators that attract TAMs and monocytes to the tumor site [196,197,198]. Not all malignant tumors have constant high chemokine levels [199]. This pro-tumor microenvironment is a good response, primarily to promote metastasis. It reduces anti-cancer proteins and cytokines [200,201,202,203,204]. Second, it plays an important role in the development, progression, and infiltration of cancer to progress fastest. This can be summarized in the following three steps: removal, balance, and escape from immune surveillance [27,179,205,206,207,208].

## 5. Macrophage Polarization

Circulating immature monocytes and bone marrow precursors released from the bone marrow differentiate into M1 or M2 macrophages [209,210,211,212,213,214,215]. Macrophages are a highly heterogenous population of cells that undergo extensive changes in their intracellular metabolism in response to environmental and inflammatory stimuli. M1/M2 classification has also been used to define macrophage polarization states, which are associated with different characteristics and functions. They can be M2-like as immunosuppressive or M1-like as immune-stimulating (initiation of the inflammatory response) [216,217,218,219]. Thus, the M1/M2 cells do not simply describe activated or inactivated macrophages, but cells expressing distinct metabolic programs. They are, in fact, two functionally different macrophages produced by in vitro differentiation of monocytes [220]. That is, they can exhibit cytotoxic activity as the first line of defense against tumor formation by directly killing tumor cells and promoting invasive and metastatic activity [27,221]. The first type is M1-like macrophage, which is classically activated and stimulated by bacterial products and alternately activated by cytokines produced by T helper 1 lymphocyte (Th1). The second is M2-like macrophages (M2a, M2b, M2c, M2d), which inhibit M1 activity and enhance metastasis [185,222,223]. The immune microenvironment plays an important role in determining monocyte/macrophage polarization and tumor progression [223,224]. In addition, within the tumor’s protective cells, macrophages are converted to immunosuppressive macrophages of TAM type M2. The different phenotypes of these two macrophages depend on their different markers, gene expression profiles of metabolic properties and microbial signals that represent macrophage polarization [113,114,115,182,185,217,225,226,227,228,229,230,231]. Well-stimulated macrophages can kill tumor cells. However, TME-stimulated TAM lacks the cytotoxic function of macrophages, but they can stimulate tumor activity, cancer-related inflammation, angiogenesis, immunosuppression, tissue remodeling, and metastasis [27,232]. Research has further shown a correlation between the degree of adiposity/obesity and chronic low-grade inflammation, macrophage recruitment, and polarization by stimulating inflammatory pathways, which can lead to increase the risk of cancer [33,112,233,234].

**Figure 2:**
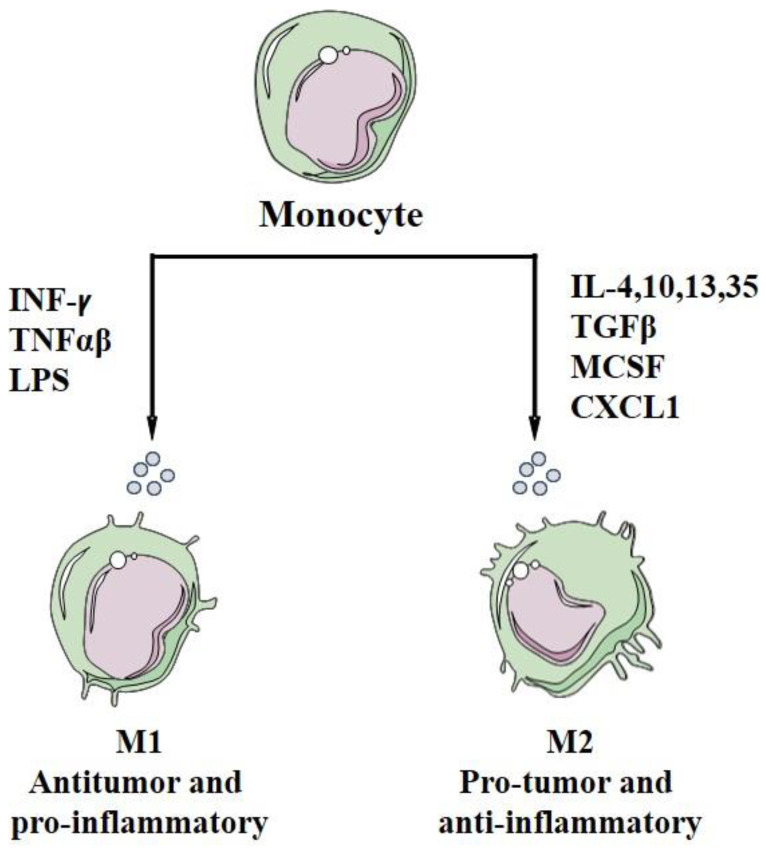
Differentiation of monocytes into macrophages M1 and M2.

**Figure 3:**
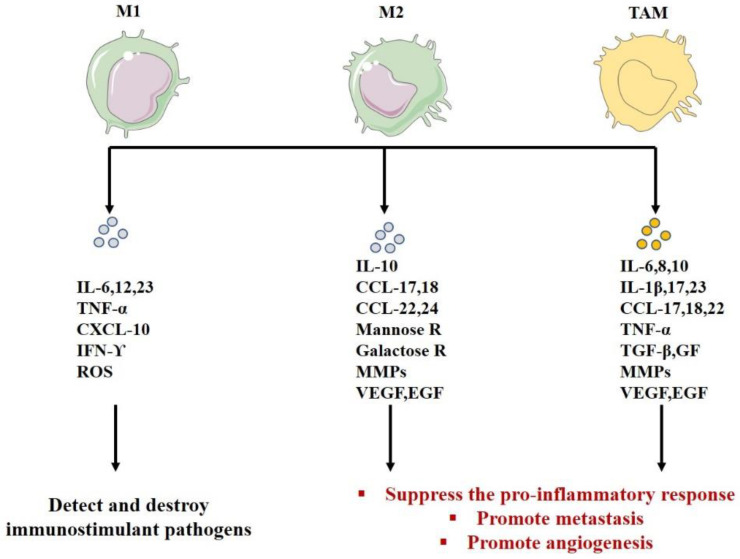
The functions of macrophages M1, M2, and TAMs according to their secretion.

M1-like macrophages activate the type I immune response responsible for protection against intracellular pathogens (antigen presenters) and tumor cells (potentially anti-tumor function) [19,222,235,236]. They express high levels of the main histocompatibility complex class II (MHC-II), CD14, CD16, CD68, CD80, and CD86 costimulatory molecules [237,238,239]. In general, this response is activated by interferon (INF)-γ, TNF-α or bacterial LPS (microbes or microbial products) [216,240] (Figure 2) and needs a recognition between a specific peptide antigen complex and the MHC of the antigen-presenting cell (usually macrophages). These activated M1s secrete immunostimulants and proinflammatory cytokines such as IFN, IL, and particularly high levels of IL-6, IL-12, IL-23, TNF-α, and chemokine ligand (CXCL)-10 [27,241,242,243,244,245] (Figure 3). M1 is characterized by anti-tumor activity because it secretes reactive oxygen species (ROS) and nitrogen intermediates that have cytotoxic effects on tumor cells [181,190,191,222,223,246,247,248,249,250,251,252,253], (Table 1). In addition, some studies have shown a link between obesity and inflammation, which stimulates chronic or mild inflammation of adipose tissue. Obesity inhibits the secretion of the anti-inflammatory adiponectin and increases the secretion of adipokines and pro-inflammatory proteins (TNF-α, IL-6, IFNγ, and TGF-β1), which enhance overexpression of MHC-II in the primary adipocytes. Such adipocytes will then behave as an antigen presenting cell (APC) to activate the adipose-resident T cells (ARTs) and stimulate the pro-inflammatory response. Finally, adipocytes can activate T cells and mobilize ATMs directly via the MHC-II pathway and consequently stimulate M1 polarization [254,255,256,257,258,259,260,261].

In contrast, M2-like macrophages are activated by immune complexes and cytokines and become the immunosuppressive phenotype [241,246,262] (Figure 2). This population is phenotypically characterized by the expression of the macrophage mannose receptor (MMR), also called CD206. These cells can also be characterized by the expression of CD163 and CD209 [239,263]. They stimulate T helper 2 response, tissue repair, and tumor progression. M2 macrophages are also characterized by hyperphagic activity, high expression of mannose receptor 1 (CD 206), galactose receptors, low levels of IL-12, high expression and secretion of IL-10 [185,217,230], angiogenic factors (VEGF, EGF), and MMPs [182,226,227,228,229].

Different subtypes of M2 macrophages can be triggered by different stimulators. They are divided into subpopulations according to the stimuli that activate them, as follows [19,173,264,265,266,267,268,269,270,271,272]:

1-Alternative M2a macrophages, also called wound healing macrophages, are induced by IL-4 and IL-13. They initiate an immune response by inhibition of Th1 and activation of Th2, thus suppressing and killing parasites. They also secrete fibrosis-promoting factors (TGF-β, IGF, etc.), IL-10, IL-1ra, promote type II inflammation, kill parasites, and express high levels of CD206, IL-1 receptor, and CCL17.

2-Regulatory macrophages M2b are activated by immune complexes, LPS, toll-like receptor (TLR) ligands, IL-1b, and physical factors (radiation). When activated, this subtype releases pro-inflammatory and anti-inflammatory cytokines as well as IL1R. In addition, they express high levels of proinflammatory cytokines (such as IL-1β, IL-6, TNF-α), and CCL1. They also express and secrete anti-inflammatory IL-10 and low levels of IL-12 to control metastasis, suppress tumor growth, and induce Th1 responses.

3-Acquired inactivated macrophages M2c are activated by IL-10, TGF-β, and glucocorticoids, which are responsible for Th1 type reactions. They strongly exhibit anti-inflammatory activities by releasing large amounts of IL-10 and secreting high levels of TGF-β. They are also involved in tissue repair and matrix remodeling.

4-M2d macrophages that represent a new M2 subgroup, also known as tumor-associated macrophages (TAMs), are activated by IL-6 and adenosines. Adenosines cause the expression of IL-10 and VEGF, leading to angiogenesis and tumor progression. M2d or TAM supports tumor progression by allowing the growth of malignant cell masses and new blood vessels.

TAM is defined as macrophages that invade tumors and are part of the TME. Depending on the type and stage of development of the tumor, it is polarized to M2 and has little ability to present antigens [273]. This subgroup of macrophages often has an anti-inflammatory phenotype. It has been identified in many studies based on biomarkers, such as CD68, CD163, CD204, and/or CD206 [212]. These cells can stimulate tumor growth and metastasis [217,247,265,274,275]. Several microenvironmental cytokines, chemokines, GFs, and other signals from tumors (such as IL-4, IL-10, TGF-1, and prostaglandin E2) and stromal cells stimulate TAM polarization into an M2 phenotype [27,170,276] (Figure 3). We noticed a high TAM density specifically in breast cancer [277] (Table 1). TAMs are promoters of the metastatic stage in the initiation and invasion of tumors [247,248,249,250,251]. They secrete various cytokines (such as IL-23, IL-17, and IL-6), which cause tumor-induced inflammation and promote tumor growth. They also secrete GFs and proteases that stimulate tumor growth and immune regulation [223,278,279]. They are mobilized and activated by several signals from TME to promote tumor progression and metastasis [181,223,252]. On the other hand, they suppress the pro-inflammatory response to tumor cells and thus allow their proliferation [223]. TAM improves vascular angiogenesis and metastasis [14,15,89,90,280]. It is also involved in the degradation of ECM by the secretion of MMP-2 and MMP-9 [113,114,115] (Table 1), which depicts the importance of ECM and macrophages in the progression of cancer [281,282]. TAM has several partially conflicting activities, both inflammatory and anti-inflammatory, immunostimulatory, anti-immunosuppressive, and tissue-destroying anti-anabolic effects [19,283,284,285,286,287,288,289,290,291,292,293]. It acts in the following three areas of the microenvironment: the infiltration area (enhancing cancer cell migration), the interstitial and perivascular areas (enhancing cancer metastasis), and/or the avascular and perinecrotic areas (where hypoxic TAMs stimulate angiogenesis) [180].

**Table 1:** Comparison between macrophages M1, M2, and TAMs.

	Macrophage M1	Macrophage M2	TAMs	References
Cell surface markers	CD14, 16, 68, 80, 86, MHCII	CD14, 163, 206, 209	CD68, 163, 204, 206	[212,237,238,239,263]
Polarization factor	Polarization of macrophages to M1 with LPS, IFN, TNF-α/γ	Polarization of macrophages to M2 with GF, CCL2, CXCL4, cytokines of Th2 (IL-4, IL-13), IL-10, IL-35, TGF-β, CXCL 1 or corticosteroids.	Same to M2s	[210,211,212,213,214,217,240,268,276]
Role	Detect, destroy immunostimulant pathogens	-Inhibit lymphocyte functions in the tumor -Suppress the pro-inflammatory response -Promote tumor progression -Promote angiogenesis-Degrade ECM	-Inhibit lymphocyte functions in the tumor -Suppress the pro -inflammatory response -Promote metastasis-Promote angiogenesis-Remodel the ECM -Suppress the adaptive immunity(M2)	[181,190,191,222,223,247,248,249,250,251,252,253]
Phenotype	Pro-inflammatory and tumor suppressor	Pro-tumor (tumor promoter)	Pro-tumor (tumor promoter)	[19,210,211,224]
Cytotoxic activity	Cytotoxic against microorganisms and tumor cells(phagocytosis)	Hyper-phagocytic (promoting debris trapping)	Hyper-phagocytic (promoting debris trapping)	[185,209,268]
Antigen presentation	High presentation potential	Low presentation potential	Low presentation potential	[216,217,218]
Effect on T lymphocyte	Produce high levels of Th1 cell stimulating cytokines	Suppress the proliferation and action of lymphocytes Th2 cells (IL-10)	Suppress the proliferation and action of lymphocytes Th2 cells (IL-10)	[19,216,217,265,266,267]
Inflammation	Stimulate inflammation	Negative control of the inflammatory response mediated by M1	Negative control of the inflammatory response mediated by M1	[216,217]
Chemokine profiles	Expressing chemokines attracting Th1 cells, such as CXCL9 and CXCL10	Release of chemokines, CCL17, CCL22, and CCL24	Release of chemokines CCL2, CXCL8, CCL18 (attract subsets of T cells lacking cytotoxic function) CCL17 and CCL22 (promote Th2 in tumors)	[19,217,225,231,283,284,285,286,287,288,289,290,291,292,293]
Immune capacity	Effective cells capable of killing tumor cells	Promote tissue remodeling and tumor progression	Promote tissue remodeling, tumor progression and metastasis	[27,209,241,243,244,245,246]
Secretion	IL-12; IL-1; ROS; IL-23 CXCL1–3; CXCL5; CXCL8–11	IL-10 autocrine circuit inhibits the expression of IL-12 and IFN-γ CCL17, 18, 22, 24 Mannose galactose ReceptorsMMP VEGF, EGF	IL-10 autocrine circuit inhibits the expression of IL-12 and IFN-γ IL-23, IL-17, IL-6 IL-8, IL-1β CCL17, 18, 22 VEGF, EGF, TNF-α, TGF-β, GFs, MMPs	[113,114,115,182,185,217,225,226,227,228,229,230,231]

Besides, adipose tissue macrophages (ATMs) are the major immune cells of the adipose tissue. In general, studies have shown that the phenotype of activated macrophage populations can change occasionally depending on the state of the microenvironment, from classic M1 (anti-tumor) to alternative M2 (protumor), and vice versa [245,294,295,296,297,298,299,300].

In the tumor-free adipose matrix, the microenvironment, by secreting soluble factors, stimulates the increase in the expression of inflammatory genes and the decrease in the secretion of anti-inflammatory IL-10 [297,301,302]. This activity results in chronic low-grade inflammation by releasing inflammatory cytokines (TNF-α, IL-6, IL-1β and CCL2) that stimulate recruitment and infiltration of macrophages and lymphocytes into the adipose tissue. Consequently, these cells are classically polarized into the M1 phenotype [295,296,297,298]. It should be noted that overweight/obesity can stimulate the repolarization of macrophages from the M2 to the M1 phenotype [245,294].

On the other hand, cancer cells in an adipose matrix secrete various cytokines that promote the repolarization of macrophages from an initial M1 profile to a subsequent M2 and TAM profile [245,299,300]. Metastasis is then the main stimulator of phenotype repolarization of macrophages from classical activation (M1) to alternative activation (M2) [168,303]. The ATMs associated with tumors gain a profile like TAMs to stimulate signaling of cytokine-receptor interactions and free fatty acid production that activates macrophages and vascular endothelial cells. The cancer-related pathways are thus overexpressed and the PMN will be more favorable to tumor growth and, consequently, metastasis [100,140,141,142,143,144,295,296,297,298,304].

Macrophages also play a major role in anti-cancer therapy resistance. Generally, the function of macrophages leads to sensitization or resistance to traditional therapy. Macrophages have different phenotypes (pro-tumor or anti-tumor) and are important regulators of homeostatic tissue and tumor microenvironments [305]. To improve the response to cytotoxic therapy, various preclinical models are used to stimulate adaptive immune responses by repolarizing macrophages (reprogram to anti-tumor phenotypic states) or blocking macrophage recruitment to tumors (reprogramming them into an anti-tumor phenotype). This phenomenon has been successful in stimulating the adaptive immune response by either neutralizing the predominant Th2-based program or by directly promoting macrophage activation [306,307,308,309]. The polarization of macrophages to M2 mediated the resistance of tumor cells to chemotherapy or radiation therapy and their inhibition showed an improved response to radiation therapy in breast cancer [310]. This transformation induces various cytokines (IL-6, TNF-α, EGF, VEGF) [251,311], to improve the proliferation and survival of malignant cells under the treatment, resulting in chemoresistance and radiation defense function [312,313,314]. Therefore, macrophages support T cell responses, limit sustained tumor growth along with cytotoxic therapy, and validate their consideration in tumor immunotherapy. Diverse drugs had some success and received clinical approval from the FDA to targeting malignant cells (with EGFR inhibitors and HER2-targeted drugs) [315,316,317] and nonmalignant cells such as cellular and molecular components of TME function.

## 6. Angiogenesis and Hypoxia

**Figure 4:**
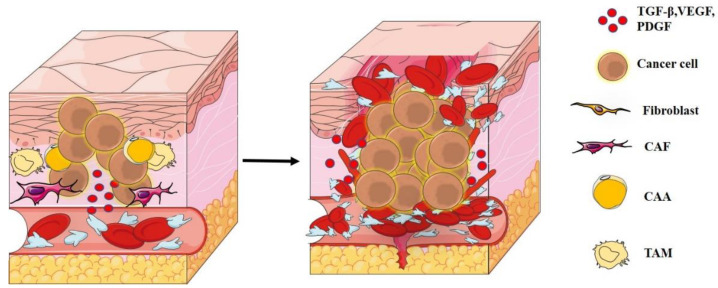
Brief description of tumor angiogenesis.

Angiogenesis is the first step involved in the appearance of metastases. In 1971, Dr. Judah Folkman suggested that tumor metastasis, progression, and enlargement (limited to 1 or 2 mm) are dependent on angiogenic regulators [179]. Various experimental studies have been conducted to show the role of alternately activated macrophages (M2, TAM) and adipocytes, which are major effectors in tumor progression [188,318,319,320,321,322,323,324] through different pathways, such as interstitial increased deposit [217,324,325,326], angiogenesis [327,328,329,330], and eventually, metastasis [100,190,217,265,291,297,301,302,304,326]. Blood and lymph networks are also involved in the supply of oxygen and nutrients to maintain cell survival and growth, or in the immune surveillance at the stage of tumor development. The angiogenic switch is a dramatic improvement in the vascular density process supported by various mediators produced by neoplastic and stromal cells. It regulates tumor vascular programming, which induces high-density angiogenesis and increases nutrient uptake and waste removal [331,332]. This switch is in the process of continuous growth of new blood vessels and remains activated almost all the time during tumor angiogenesis. Existing intra-tumor blood vessels and infiltrative foreheads form a cluster of new blood vessels surrounding the tumor. Such vessels are mostly ineffective, twisted or permeable [231,322,333,334,335,336]. In general, angiogenesis is activated by tumors (at a particular tumor size) to promote metastasis [322,324,337] (Figure 4). The formation of a complete vasculature is considered a complex process involving many cell types, often with overlapping functions that affect tumor outcomes, such as TAM and CAA cells, which express many factors that intervene in this process, citing the following:

1-Angiogenesis-promoting factors such as TGF-β, VEGF, and PDGF that increase the density of microvessels, reverse the effects of angiogenesis, promote macrophages, and stimulate invasion into tumors and metastases [19,188,200,318,319,320,321,322,323,324]. On the other hand, their loss leads to the normalization of blood vessels [299,300,321,338,339].

2-Cytokines, GFs, and activated myofibroblasts that stimulate the deposition of fibers in the interstitium and create a coagulation-promoting state, improving the formation of indirect blood vessels [217,324,325,326].

3-MMPs such as MMP2, MMP7, MMP9, and MMP12 that mediate bioavailability (MMP9) and activate TGF-β (MMP2) to promote angiogenesis [17,37,108,109,110,120,121,122,140,141,142,143,144,306,312,320,321].

4-Various chemokines CXCL1, CXCL8, CXCL12, CXCL13, CCL2, CCL5 [100,265,297,301,302,304,326].

Besides, hypoxia, a phenomenon associated with hypoxic partial pressure, is usually an important factor in improving angiogenesis. Cell proliferation in cancer is known to require a lot of energy, and therefore oxygen, to progress. When intratumorally, O_2_ is reduced, the HIF-1 protein then helps form new blood vessels for O_2_ supply. HIF-1 proteins act as transcription factors in all tissues and are regulated by O_2_ deficiency [322,323]. This protein was discovered in 1992 by Gregg L. Semenza, who studied the EPO gene. Tissue with hypoxia is usually treated by stimulating EPO production, which induces red blood cell production, resulting in improved transport of O_2_ to the target tissue. The low intratumor oxygen level helps HIF-1 proteins form new blood vessels for oxygen supply [323,324]. 

On the other hand, advanced TME is characterized by a strong metabolism and a high tumor growth level [325,326]. In solid tumors, peripheral blood vessels become hypoxic due to inadequate oxygen supply. In contrast, primary tumor cells are highly flexible and can quickly adapt to hypoxic content or migrate to blood vessels to promote tumor growth [323,324,326,327,328]. The tumors then show a strong tendency to metastasize. In addition, hypoxia polarizes macrophage phenotypes to a protumor profile M2 through lactate secretion. Lactic acid is released from anaerobic glycolysis of tumor cells in the region of hypoxic partial pressure [322,329]. Hypoxia is also a vital regulator of immune recruitment, by promoting the behavior of malignant tumors, promoting glycolysis and cell differentiation, inhibiting apoptosis, and reducing therapeutic efficacy [275,330,331,332,333]. In hypoxia, chemokinesis secretions (CCL2) recruit macrophages to the hypoxic region of the tumor, where they accumulate. Their accumulation in the hypoxic region leads to aggressive tumor behavior [179,302,334,335]. Normally, due to hypoxia and overexpression of HIF-1, TAM inhibits effector T cells by secreting immunosuppressive cytokines (IL-10) [336] and stimulating angiogenesis around cancer necrosis and angiogenesis. Moreover, it expresses VEGF and TAM in the region of cancer [188,337,338] and produces various cytokines (such as IL-23 and IL-17 that cause tumor-induced inflammation and promote tumor growth, GF, and proteases to enhance tumor growth, angiogenesis, immune regulation, and metastasis) [223,278,279]. Macrophages and adipocytes can secrete VEGF, but in other cases, the VEGF becomes biologically available through the production of MMP9, which degrades ECM, resulting in collagen-encapsulated VEGF [37,107,108,109,110].

Radiotherapy, chemotherapy, and immunotherapy are the mainstays of cancer treatment. Immunotherapy strategies targeting nonmalignant cells have been developed, including the following: (a) antiangiogenic drugs (neutralizing antibody targeting VEGF) [340,341,342,343], (b) drugs targeting TAMs (emactuzumab), which contribute to chemoresistance by inducing prosurvival and antiapoptotic signals in cancer cells [344,345], (c) anti-cachexia drugs (thalidomide and megestrol acetate to downregulate IL-6 and TNF-α production) [346], (d) adoptive cellular immunotherapy (by transferring specific immune cells to the tumor-bearing host) [347], (e) antibodies (rituximab and trastuzumab) [348,349], (f) small-molecule inhibitors (synergistic with other immune-oncology therapies to suppress tumors by targeting macrophage-associated molecules) [350], (g) immune checkpoint inhibitors (blocking immunocytokines and the PD-1/PD-L1 pathways with inhibitors to enhance the cytotoxic function of T cells) [351] and (h) multiple agents targeting Th2 cytokines and their receptors (targeting IL-4, IL-13, IL-4Rα) [352,353].

## 7. Conclusions

The microenvironment of inflammatory adipose tissue is rich in multiple types of cells, including inflammatory cells and stromal cells. Adipocytes and macrophages indirectly work together to create a classic low-grade, anti-tumor inflammatory condition. This inflammation results from a pro-inflammatory dialogue between adipocytes and immune cells. However, there are several mechanisms in adipose tissue that produce chronic low-grade inflammation. Adipocytes activate the inflammatory response, increase adipocyte IL-10, weaken inflammatory ATM polarization, activate ART proliferation, stimulate ATM accumulation and polarization, and escalate fat inflammation as an APC. Adipocytes could mimic the role of macrophages. These same cells in EMT create an anti-inflammatory phenotype and prevent the anti-tumor immune response cells from reaching the cancer site. Adipocytes, macrophages, and tumor cells can then regulate several important signaling pathways to create a microenvironment that promotes cancer progression and metastasis. According to current studies, cancer cells degrade ECM, activate myofibroblasts, mobilize immune cells into tumors, and secrete various cytokines and GFs that differentiate, activate, and polarize macrophages into TAM and M2. These cells are preferably destined to meet the needs of cancer cells in a pro-tumor function (M2) instead of a pro-inflammatory anti-cancer function (M1) and enhance their malignant behavior towards tumor metastasis. Macrophages play an important role in tumors. Depending on the mode of activation, they can promote tumor growth, suppress local immunity, attack tumor cells, and even maintain tumor immunity. To decipher such complex functions requires a good understanding of how different macrophage subsets of human tumors interact with adipocytes in overweight and obese people. For this reason, cancer is a malignant disease that reverses the normal function of protective cells to promote metastasis that normally occurs in most patients. On the other hand, the details of the molecules and cells involved in promoting metastasis can be more complex than we expect. Recently, various three-dimensional (3D) in vitro models used the macrophage phenotype of stromal cells to describe regulators involved in ECM degradation, metastasis, recruitment, and macrophage polarization in the adipose matrix. Cells develop in an organized 3D matrix whose behavior depends on their interaction with adjacent cells and the ECM. A 3D culture is an important tool for better understanding changes and interactions between macrophage phenotypes during molecular signaling of malignant transformation and metastasis.

## Data Availability

Not applicable.

## Abbreviations

ASC: adipose stem cell; APC: antigen presenting cell; ART: adipose-resident T-cell; ATM: adipose tissue macrophage; CAA: cancer-associated adipocyte; CAF: cancer-associated fibroblast; MDSC: myeloid-derived suppressor cell; M-MDSC: myeloid MDSC; G-MDSC: granulocytic MDSC; CCL: chemokine ligand; ECM: extracellular matrix; EGF: epidermal growth factor; EMT: epithelial-mesenchymal transition; FN: fibronectin; GF: growth factor; HIF: hypoxia inducible factor; IL: interleukin; LOX: lysyl oxidase; MMP: matrix metalloproteinase; O_2_: dioxygen; PMN: pre-metastatic niche; ROS: reactive oxygen species; TAM: tumor-associated macrophage; TGF: transformative growth factor; Th1: T helper1 lymphocyte; TME: tumor microenvironment; TNF: tumor necrosis factor; WAT: white adipocyte tissue; EPO: Erythropoietin.
